# Enhancing the Quality of Care in Long-Term Care Settings

**DOI:** 10.3390/ijerph19031409

**Published:** 2022-01-27

**Authors:** Reena Devi, Adam Gordon, Tom Dening

**Affiliations:** 1 School of Healthcare, University of Leeds, Leeds LS2 9JT, UK; 2 Nurturing Innovation in Care Home Excellence in Leeds (NICHE-Leeds), Leeds, UK; 3 School of Medicine, University of Nottingham, Nottingham NG7 2RD, UK; Adam.Gordon@nottingham.ac.uk (A.G.); tom.dening@nottingham.ac.uk (T.D.); 4 National Institute of Health Research (NIHR) Applied Research Collaboration-East Midlands (ARC-EM), Nottingham, UK

The quality of care in long-term care settings is a concern felt across the world given the growing number of dependent older people [1]; a population whose health and care needs are increasing over time [2]. This Special Issue set out to attract research focused around enhancing the quality of care in this setting. Since 2019, we have received articles at a regular pace and this Special Issue offers a collection of 22 manuscripts focused on a wide variety of topics. This Special Issue will now close, and in this closing editorial we summarise the content and share our thoughts on future research in this area.

Research included took place around the world (UK, Australia, Taiwan, The Netherlands, Belgium, Korea, USA, Spain, Brazil, Germany, and Italy), used a range of methods, and examined quality of care from different angles. Five papers focused on workforce, adding important evidence around supporting staff with training [3], the influences on job competency, satisfaction, and intention to stay in work [4,5], staff burnout [6], and the relationship between staff and organisation with quality of care [7]. Evidence aimed at teams who work with the sector to improve quality of care was also included. One paper presented a tool containing questions designed to help initiate conversations between innovators and care home staff [8], and another paper outlined essential learning directed at teams applying a Quality Improvement Collaborative tool in this context [9]. This Special Issue also comprises intervention studies, with interventions aimed at addressing depressive symptoms in nursing home residents [10,11], adjustment for new residents [12], social and psychological support [13], and loneliness and isolation [14]. Other studies present evidence which developed and tested quality indicators [15,16], and tools which capture the experience of quality from a resident’s perspective [17,18], and assess partnership working between staff and families [19]. We also included studies that investigated factors associated with older people’s experiences, such as the association between length of stay and end of life care [20], dry eyes or ocular lubricants with medication use, dementia, frailty and dry eyes [21], resident characteristics and their palliative care service use and comfort in the last week of life [22] and causes of infection-related hospitalizations [23]. Finally, the issue also includes a systematic review describing the current evidence base of care home research conducted in Brazil [24].

The articles published in this Special Issue on enhancing care in long term care offer an array of insights, contributions and perspectives from different angles. This highlights that enhancing quality is a complex issue, one that requires relevant stakeholders to take into consideration different types of knowledge. For example, there is a need to understand causes and associations of poor and good quality, the needs of the workforce, effective interventions that have undergone robust testing, and tools which can help to effectively guide implementing evidence into practice and measure the effectiveness of change. As editors of this Special Issue, we would have liked to see more evidence uncovering how to stimulate and sustain change in care home practice. From our experience of working in this area, care homes within their day to day “business as usual” activities have made progress with initiating changes. This activity though is rarely captured in the academic literature. We suggest that future researchers bring to the centre stage evidence around the specific processes and organisational structures that help care homes to successfully initiate and sustain improved outcomes in this setting.

We are pleased to offer interesting papers from across the world in this important field and bring them together in this way. The regular pace at which we received submissions to this Special Issue indicates significant interest and relevance of the issue. We hope that readers will both enjoy and use these findings in their own research and practice.
